# Pandemic Influenza Due to pH1N1/2009 Virus: Estimation of Infection Burden in Reunion Island through a Prospective Serosurvey, Austral Winter 2009

**DOI:** 10.1371/journal.pone.0025738

**Published:** 2011-09-29

**Authors:** Koussay Dellagi, Olivier Rollot, Sarah Temmam, Nicolas Salez, Vanina Guernier, Hervé Pascalis, Patrick Gérardin, Adrian Fianu, Nathanael Lapidus, Nadège Naty, Pablo Tortosa, Karim Boussaïd, Marie-Christine Jaffar-Banjee, Laurent Filleul, Antoine Flahault, Fabrice Carrat, Francois Favier, Xavier de Lamballerie

**Affiliations:** 1 GIS CRVOI, Centre de Recherche et de Veille sur les Maladies Emergentes dans l'Océan Indien, Saint-Denis, La Réunion; 2 Institut de Recherche pour le Développement (IRD), Marseille, France; 3 Centre d'Investigation Clinique-Epidémiologie Clinique (CIC-EC) de La Réunion (INSERM/CHR/Université/URMLR), Centre Hospitalier Régional, Saint-Pierre, La Réunion; 4 Ecologie Microbienne (UMR 5557) CNRS-Université de Lyon, Lyon, France; 5 Unité des Virus Emergents (UMR-S 190), IRD-Université de la Méditerranée, Marseille, France; 6 Epidémiologie des Maladies Infectieuses et Modélisation (UMR-S 707), INSERM-Université Pierre et Marie Curie, Paris, France; 7 Chaire mixte CNRS INEE-Université de La Réunion, Saint Denis, La Réunion; 8 Département de Microbiologie, Centre Hospitalier Régional, Saint Denis, La Réunion; 9 Cellule Interrégionale d'Epidémiologie (CIRE) Réunion-Mayotte (InVS), Saint-Denis, La Réunion; 10 Ecole des Hautes Etudes en Santé Publique, EHESP, Rennes-Sorbonne Paris Cité, Paris, France; Statens Serum Institute, Denmark

## Abstract

**Background:**

To date, there is little information that reflects the true extent of spread of the pH1N1/2009v influenza pandemic at the community level as infection often results in mild or no clinical symptoms. This study aimed at assessing through a prospective study, the attack rate of pH1N1/2009 virus in Reunion Island and risk factors of infection, during the 2009 season.

**Methodology/Principal Findings:**

A serosurvey was conducted during the 2009 austral winter, in the frame of a prospective population study. Pairs of sera were collected from 1687 individuals belonging to 772 households, during and after passage of the pandemic wave. Antibodies to pH1N1/2009v were titered using the hemagglutination inhibition assay (HIA) with titers ≥1/40 being considered positive. Seroprevalence during the first two weeks of detection of pH1N1/2009v in Reunion Island was 29.8% in people under 20 years of age, 35.6% in adults (20–59 years) and 73.3% in the elderly (≥60 years) (P<0.0001). Baseline corrected cumulative incidence rates, were 42.9%, 13.9% and 0% in these age groups respectively (P<0.0001). A significant decline in antibody titers occurred soon after the passage of the epidemic wave. Seroconversion rates to pH1N1/2009 correlated negatively with age: 63.2%, 39.4% and 16.7%, in each age group respectively (P<0.0001). Seroconversion occurred in 65.2% of individuals who were seronegative at inclusion compared to 6.8% in those who were initially seropositive.

**Conclusions:**

Seroincidence of pH1N1/2009v infection was three times that estimated from clinical surveillance, indicating that almost two thirds of infections occurring at the community level have escaped medical detection. People under 20 years of age were the most affected group. Pre-epidemic titers ≥1/40 prevented seroconversion and are likely protective against infection. A concern was raised about the long term stability of the antibody responses.

## Introduction

In April 2009, the first cases of acute respiratory infections caused by a novel triple-reassortant influenza virus, pH1N1/2009v, occurred in Mexico and the United States [1]. The rapid spread of infection to other continents led the World Health Organization (WHO) to declare on 11 June 2009 that a pandemic of pH1N1/2009v influenza was under way, which raised major international concern about the risk of high morbidity and lethality and the potential for severe socio-economic impact. Actually, the potential impact of this first third-millenium influenza pandemic has been revisited downwards as morbidity and case-fatality rates were less severe than initially anticipated [2]. Illness surveillance data do not allow to an accurate estimate of the true influenza infection rate, as a substantial proportion of infections are asymptomatic or mild [3]. Serological surveys can overcome this limitation, but must take into account that a significant proportion of the population that exhibited cross-protective antibody titers before circulation of the pH1N1/2009v [4]. This so-called “baseline immunity” has to be subtracted from the seroprevalence observed after the pandemic wave, to determine seroincidence in serosurveys [5]–[8]. However, except for few studies [9]–[11], most of these serosurveys did not use serial measurements in the same person, which allows for a better understanding of antibody kinetics and the dynamics of infection within individuals and communities.

Reunion Island (805,500 inhabitants) is a French overseas department located in the southwestern Indian Ocean, 700 km east of Madagascar and 200 km southwest of Mauritius. The first imported case of pH1N1/2009v was identified on 5^th^ July 2009 (week 29) in a traveller returning from Australia. The first case indicating community transmission was detected on 21^st^ July (week 30). pH1N1/2009v became the predominant circulating influenza virus within four weeks of its first detection, its activity peaked during week 35 (24–30 August) and ended at week 38 [12]. Contrary to initial fears, the health care system was not overwhelmed, as morbidity and mortality rates were lower than predicted [12]–[14].

In order to assess at the community level, the actual magnitude of the pH1N1/2009v pandemic and the extent of the herd immunity acquired after passage of the epidemic wave, a prospective population serosurvey was conducted in Reunion Island during the passage of the epidemic wave in the 2009 austral winter season (July–December 2009): prevalence of infection was assessed on a weekly basis and seroconversion rates were measured using paired sera.

## Methods

### Sample cohorts and collection

The CoPanFLu-RUN was part of the CoPanFLu international project, a consortium between the French National Institute of Health and Medical Research (INSERM), the Institute of Research for Development (IRD) and the Mérieux Fondation under the promotion of the School of Advanced Studies in Public Health (EHESP). To enable the rapid implementation of the study in anticipation of the imminent spread of the pandemic wave, we used a pre-existing sample of 2442 households established in October 2006 for the investigation of the Chikungunya outbreak (SEROCHIK) and updated in May 2008 throughout a follow-up telephone survey (TELECHIK) on a basis of 1148 households [15], [16]. We took special attention to select households representing a wide range of geographic locations in order to minimize the repartition bias.

The inclusion phase started on July 21^st^ (week 30) and was continued up to week 44, throughout the epidemic wave and beyond. A first serum sample (sample 1) was obtained from each household member. An active telephonic inquiry was then conducted twice a week to record symptoms compatible with influenza-like illness (ILI) occurring in households. Report of ILI (fever ≥37.8°C associated with any respiratory or systemic symptom) led to three consecutive visits of a nurse to the incident case-dwelling (on day 0, +3 and +8 post-report) to record symptoms and collect nasal swabs from all family members (for qRT-PCR detection of pH1N1/2009v. At week 45, the active inquiry was discontinued and a second (post-epidemic) serum sample (sample 2) was obtained (weeks 45–52) to determine seroconversion rates. Sera were aliquoted and stored at −80°C.

### Ethical considerations

The protocol was conducted in accordance with the Declaration of Helsinki and French law for biomedical research (N° ID RCB AFSSAPS: 2009-A00689-48) and was approved by the local Ethics Committee (Comité de Protection des Personnes of Bordeaux 2 University). Every eligible person for participation was asked for giving their written informed consent.

### Laboratory procedures

#### Viral genome detection by RT-PCR

Viral RNA was extracted from 140 µL of nasal swab eluate using the QIAamp Viral RNA kit (Qiagen) and processed for detection by TaqMan qRT-PCR targeting the heamagglutinin HA gene (SuperScript III Platinum one-step qRT-PCR system, Invitrogen) according to the recommendations of the Pasteur Institute (Van der Werf S. & Enouf V., SOP/FluA/130509). Confirmed pH1N1/2009v infection was defined as a positive qRT-PCR detection of the HA gene in at least one nasal swab.

#### Hemagglutination inhibition assay (HIA)

A standard hemagglutination inhibition technique was adapted to detect and quantify pH1N1/2009v antibodies [17]. The antigen was prepared by diluting a non-inactivated cell culture supernatant producing a pdm H1N1v strain (strain OPYFLU-1 isolated from a young patient returning from Mexico in early May 2009) [18]. Briefly, the virus was propagated onto MDCK cells under standard conditions. The last passage (used for antigen preparation) was performed in the absence of trypsin and ht-FBS. The supernatant was collected at day seven p.i. clarified by centrifugation at 800× g for 10 min at room temperature, aliquoted and conserved at −80°C. The hemagglutinating titer of the non inactivated viral antigen was immediately determined under the HIA format described below. The dilution providing 5.33 hemagglutinating units in a volume of 25 µL was used for subsequent HIA. Sera were heat-inactivated at 56°C for 30 min prior to use. Sequential twofold dilutions in PBS (1/10 to 1/1280) in volumes of 25 µL were performed and distributed in V-bottom 96 well microplates. Human red blood cells (RBC) were used for hemagglutination experiments. Detection and quantification of antibody to pH1N1/2009v was performed as follows: 25 µL of virus suspension was added to the serum dilution (25 µL) and incubated for 1 hour at room temperature. Each well was then filled with 25 µL of a 1% RBC suspension in PBS (v/v: 0.33%), followed by another 30 min incubation at room temperature. The HIA titer was determined as the last dilution providing clear inhibition of hemagglutination. All experiments were performed in the presence of the same negative and positive controls, the latter including sera with 1/40, 1/80, 1/160 and 1/320 antibody titers.

### Outcome measures

The results reported in this study were based only on serological analysis of paired sera. For the sake of analysis, four successive phases were identified throughout the pandemic wave: phase A (weeks 30–31) corresponded to early epidemic time, phase B (W32–39) to the epidemic unfolding, phase C (W40–44) to the immediate post-epidemic stage and phase D (W45–52) to the late post-epidemic stage. Seropositivity was defined as a HIA titer of 1/40 or more. The baseline-proxy seroprevalence rate was estimated on serum samples collected in phase A. The cumulative incidence rate of infection measured the raise between the raw seroprevalence rate at any given time during the epidemic phases (S2pi) and the age-specific baseline-proxy seroprevalence rate (S1pA) (s2_pi_-s1_pA_). Seroconversion was defined as a shift from seronegative at inclusion (sample 1: HIA <1/40) to seropositive on follow-up (sample 2: HIA ≥1/40), or for sera tested seropositive on inclusion as a four-fold increase of HIA titers between sample 1 and sample 2 paired sera. We also calculated the proportion of sera that tested seropositive in sample 1 for which the HIA titer decreased four-fold and passed under the cut-off value of 1/40 in sample 2. We considered this proportion as a “seronegation” rate.

### Statistical analysis

The sample size was calculated for identifying risk factors in the prospective cohort study. Considering on average three individuals per household, an intra-household correlation of 0.3, a power greater than 80% could be obtained with a sample size of 840 comprising 2500 individuals, assuming exposure levels ranging from 10% to 90% and a relative risk greater than 1.3. With 2,500 subjects, the study allowed 1–2% absolute precision around the estimated values for seroconversion rates. Data entry used EpiData version 3.1 (The Epidata Association, Odense, Denmark). SAS version 9.1 (SAS Inc., Cary, NC, USA) was used for statistical analysis. The characteristics of the study cohort were compared to those of the population of Reunion Island and a Chi2 test (or Fisher's exact test when non applicable) was used to analyse differences in age, sex and geographic location. Cumulative incidence rates of infection (i.e. seroincidence) and seroconversion rates were standardized according to the age structure of the community (French National Institute for Statistics and Economical Studies (INSEE) source).

Baseline-proxy seroprevalence, cumulative incidence rates of infection, as well as seroconversion and seronegation rates, were expressed as percentages. Cumulative reverse distribution curves were used to show the distribution of antibody titers. In all tests, a *P* value<0.05 was considered significant.

We estimated 95% confidence intervals (CIs) of proportions by using a cluster bootstrap technique with 1000 re-samples [19]. After bootstraping, we used an ANOVA model to compare mean cumulative incidence proportions between pandemic phases, within each age group. We used an alternating logistic regression model (ALR) with an exchangeable log Odds Ratio (OR) to test the intra-household correlation-adjusted association between factors and the seroconversion outcome.

Data were analysed with respect to subject age. Initially, four age groups were considered: the children and adolescents (<20 yrs), young adults (20–39 yrs), middle-age adults (40–59 yrs), and elderly adults (≥60 yrs). As the cumulative incidence of infection of the second and third groups were very close, both groups were merged into one adults group (20–59 yrs). Therefore we refer further in our study to three age groups: children and adolescents (<20 yrs), adults (20–59 yrs), elderly (≥60 yrs).

## Results

### Description of the CoPanFlu-RUN cohort

**Figure 1:**
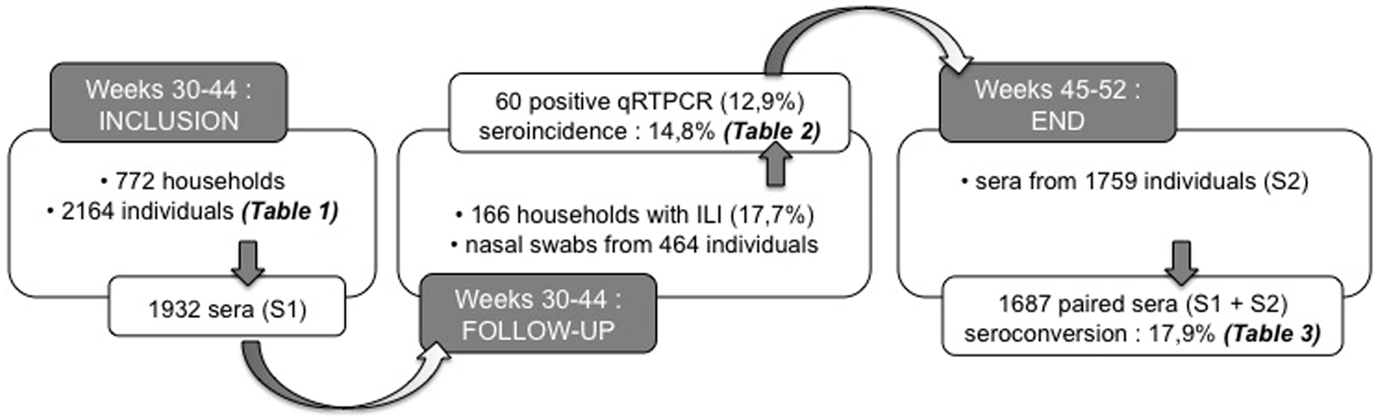
The cohort profile and major outcomes. Figure 1 details the three phases of the protocol: i) inclusion (weeks 30–44) and serum samples S1 collection; ii) follow up for detection of ILI in households, qRT-PCR on nasal swabs and estimation of cumulative seroincidence rates; iii) end of the study (weeks 45–52) and samples S2 collection. HIA on paired sera (S1+S2) allowed estimating seroconversion rates.

**Figure 2:**
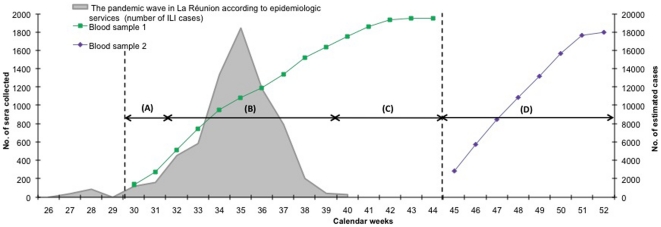
Synoptic view of CoPanFlu protocol implementation. Green and violet lines represent the number of blood samples collected from the cohort, on inclusion (weeks 30–44) and at the end of the study (weeks 45–52) respectively (Y axis at the left: number of samples collected). Shaded area represents the profile of the epidemic wave in Reunion Island according to the local epidemiology surveillance unit (Y axis at the right: estimated number of symptomatic influenza cases occurring in the island); X axis: calendar weeks.

A total of 2,164 individuals from 772 households were enrolled between weeks 30 and 44 in the CoPanFlu-RUN cohort, allowing the collection of 1,932 sera at inclusion (sample 1). During this period, 136 households (17.7% of households) containing 464 individuals (21.4% of individuals) reported at least one case of ILI. Sixty subjects among the 464 individuals (12.9%, belonging to 33 households [24.3%]) were qRT-PCR positive, which documented the pH1N1/2009v infection. No positive qRT-PCR could be detected after week 37 and no ILI was reported after week 40, the end of the epidemic wave. The second follow up serum sample (sample 2) was obtained for 1,759 subjects at least five weeks after the end of the epidemic wave (weeks 45–52) which allowed the constitution of a serobank of 1,687 paired-sera. The profile of the cohort and the major outcomes are displayed in Figure 1. Details on inclusions and serum sample timing with respect to the circulation of pH1N1/2009v over the island are provided in figure 2. The socio-demographic and space-time characteristics of the cohort are detailed in Table 1. Compared to the community of Reunion Island, the sample of 1,687 individuals for whom paired-sera were available, was older (<20 yrs: 27% vs 35%, and ≥60 yrs: 17,9% vs 11,3%) and composed of a slight excess of females (54.1% vs 51.5%). The imbalance was due to a deficit in subjects aged under 40 years, reflecting men at work and the fact that parents declined the second serum for children younger than five.

**Table 1:** Socio-demographic and space-time characteristics of the COPanFlu-RUN cohort subjects compared to the community, Reunion Island, 2009.

Characteristics of individuals	enrolled in the study	sampled at inclusion	sampled at inclusion and follow-up*	Community**
**Age group**				
<20 years	697 (32.2%)	535 (27.7%)	458 (27.1%)	35.0%
20–39 years	495 (22.9%)	471 (24.4%)	401 (23.8%)	27.9%
40–59 years	614 (28.4%)	582 (30.1%)	526 (31.2%)	25.8%
≥60 years	358 (16.5%)	344 (17.8%)	302 (17.9%)	11.3%
**Gender**				
Male	1,003 (46.3%)	889 (46.0%)	774 (45.9%)	48.5%
Female	1,161 (53.7%)	1,043 (54.0%)	913 (54.1%)	51.5%
**Household location**				
Eastern	425 (19.6%)	352 (18.2%)	281 (16.7%)	14.8%
Northern	305 (14.1%)	274 (14.2%)	217 (12.9%)	23.9%
Western	628 (29.0%)	578 (29.9%)	518 (30.7%)	25.5%
Southern	806 (37.3%)	728 (37.7%)	671 (39.8%)	35.8%
**Time of inclusion (weeks)**				
W 30–31	302 (14.0%)	269 (13.9%)	249 (14.8%)	-
W 32–39	1493 (69.0%)	1344 (69.6%)	1174 (69.6%)	-
W 40–44	367 (17.0%)	319 (16.5%)	264 (15.6%)	-
**Total**	2,164	1,932	1,687	805,500

Data are numbers (percentages);

*paired sera;

**French National Institute for Statistics and Economical Studies (INSEE) source.

### Baseline-proxy seroprevalence rates to pH1N1/2009

Baseline-proxy (∼pre-epidemic) HIA titers to the pH1N1/2009v were measured on sample 1 (Table 2), obtained from 249 subjects (103 households) recruited at the very beginning of the investigation during weeks 30 and 31 (phase A, Figure 2), when the epidemic activity in the cohort was still very low. Age distribution in this group was similar to that of the whole cohort (data not shown). The overall, the baseline-proxy seroprevalence rate (HIA ≥1/40), over all ages, was 43.4% (95%CI: 37.4%–49.6%). However the majority of positive sera had low antibody titers, at the cut off value for confirmation (i.e. = 1/40). The proportions of sera with HIA titer >1/40 were 0%, 3.0% and 24.6% in the young, middle-aged and older age groups respectively. These results indicate that pre-epidemic baseline antibody cross reactivity was stronger in the elderly (≥60 yrs) and weaker in children and adolescents (<20 yrs) and adults (20–59 yrs), with highly significant differences between age groups (*P*<0.0001).

**Table 2:** Baseline-proxy seroprevalence and cumulative incidence of infection by 2009 pandemic influenza A H1N1 virus (pH1N1/2009) according to age and to epidemic phase, CoPanFlu-RUN cohort, Reunion Island, 2009.

Age group	Pandemic phase(weeks)	Period study(No. of blood samples)	Seropositivity (HIA* titer ≥1/40)		*P* value
			Bp seroprevalence rate^a^	Cumulative incidence rate^b^	
<20 years	W32–39	Inclusion/follow up (325)	29.8% (19.5% to 42.7%)	28.3% (22.2% to 34.0%)	<0.0001
	W40–44	Inclusion/follow up (76)	29.8% (19.5% to 42.7%)	61.0% (54.2% to 66.6%)	
	W45–52	End of the study (458)	29.8% (19.5% to 42.7%)	42.9% (38.2% to 47.2%)	
20–59 years	W32–39	Inclusion/follow up (639)	35.6% (27.9% to 44.1%)	6.3% (2.4% to 10.3%)	<0.0001
	W40–44	Inclusion/follow up (156)	35.6% (27.9% to 44.1%)	45.8% (39.4% to 51.6%)	
	W45–52	End of the study (927)	35.6% (27.9% to 44.1%)	13.9% (10.4% to 17.6%)	
≥60 years	W32–39	Inclusion/follow up (210)	73.3% (61.0% to 82.9%)	−8.6% (−15.8% to −2.0%)	<0.0001
	W40–44	Inclusion/follow up (32)	73.3% (61.0% to 82.9%)	20.4% (11.5% to 26.7%)	
	W45–52	End of the study (302)	73.3% (61.0% to 82.9%)	−10.7% (−16.0% to −5.2%)	
All ages	W32–39	Inclusion/follow up (1,174)	43.4% (37.4% to 49.6%)	12.3% (9.2% to 15.4%)	-
	W40–44	Inclusion/follow up (264)	43.4% (37.4% to 49.6%)	48.2% (44.2% to 52.3%)	
	W45–52	End of the study (1,687)	43.4% (37.4% to 49.6%)	21.3% (18.7% to 23.8%)	

Data are numbers, percentages (95% confidence intervals) and ANOVA test *P* value for comparison of mean cumulative incidence proportions between pandemic phases, in each age groups. Distributions were estimated by non parametric cluster bootstrap technique with 1000 resamples of households. W30–31: early epidemic phase (baseline-proxy); W32–39: full development of the epidemic wave; W40–44: immediate post-epidemic phase; W45–52: late post-epidemic phase.

*HIA titer: Hemagglutination inhibition assay titer.

a Bp (baseline-proxy) seroprevalence rates were estimated on weeks 30–31 in each age group.

b Cumulative incidence rates measured the raise between raw seroprevalence rates and age-specific baseline-proxy seroprevalence rate. In the group “All ages”, cumulative incidence rates were standardized according to age structure of the community.

### Cumulative incidence rates of pH1N1/2009 influenza during and after passage of the pandemic wave

**Figure 3:**
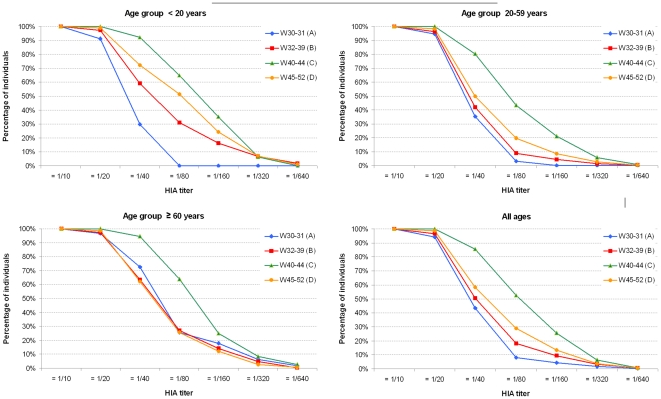
Reverse cumulative distribution HI curves according to age and to epidemic phases. baseline-proxy, early epidemic phase A (W30–31); per-epidemic phase B (W32–39); early post-epidemic phase C (W40–44) and late post epidemic phase D (W45–52).

The reverse cumulative distribution curves of HIA titers are displayed for each age group and for the whole cohort on Figure 3. The proportion of seropositive sera (HI ≥1/40) steadily increased during the epidemic unfolding (phase B, W32–39) and in immediate post epidemic period (phase C, W40–44) when it reached its maximum level, then declined in the late post epidemic period (phase D, W45–52). This decline was significant enough to return the reverse cumulative distribution curve to baseline levels in the elderly. The cumulative incidence rates, obtained after subtraction of the age-specific baseline-proxy seroprevalence from the raw seroprevalence at each phase of the epidemic are shown in Table 2 (note that the cumulative incidence rates of infection represented for the group “all ages” were standardized according to age structure of the community). The cumulative incidence rates were much higher in children and adolescents (<20 yrs), indicating very active transmission of infection within this age group. As mentioned earlier, cumulative incidence rates peaked in phase C (W40–44), and then declined indicating some lability of the humoral immune response against the pH1N1/2009v. The age-related difference observed in the incidence rates was highly statistically significant (*P*<0.0001).

To estimate more appropriately the decline of antibody titers occurring after the peak of the humoral response to the pH1N1/2009v, we considered paired-sera from the group of 264 subjects for whom the first serum sample (sample 1) was obtained just after the epidemic wave (phase C, W40–44), and the corresponding second sample was collected at the end of the survey (phase D, W45–52). Seronegation rates were 27.0% (61/226) for all age groups, 17.4% (12/69) in children and adolescents (<20 yrs), 32.3% (41/127) in adults (20–59 yrs) and 26.7% (8/30) in the elderly (≥60 yrs). Differences between the seronegation rates according to age were statistically weakly significant (P = 0.0671).

### Seroconversion rates in the cohort and in individuals with documented (qRT-PCR positive) pH1N1/2009 infection

We then considered the 1687 individuals for whom paired sera were available and we measured the seroconversion rates according to age and to the time of first serum sample collection (phase A, B or C). Criteria of seroconversion were defined in the method section. As shown in table 3, there was a sharp decline in seroconversion rates across all the age groups, depending on whether participants were enrolled during phase A, phase B, or phase C (*P*<0.0001). To interpret these data, one should remember that antibodies at seroprotective levels (HIA ≥1/40), in serum samples 1 collected during the per epidemic phase B or early post epidemic phase C could represent either base line cross reactive antibodies or rising pH1N1/2009 specific antibodies due to a recent or ongoing infection. This ambiguity could lead to underestimation of the seroconversion rate for subjects enrolled in phases B and C. In order to solve this ambiguity, we specifically considered the group of 249 subjects in whom cross reactive antibodies were detected at the time of phase A (W30–31). The seroconversion rate of this group is the most indicative of the exposure of individuals to the whole epidemic wave. It was the highest (63,2%, *P*<0.0001) in children and adolescents (<20 yrs), and still significantly high in adults (39.4%, *P*<0.0001).

**Table 3:** Seroconversion rates to 2009 pandemic influenza A H1N1 virus (pH1N1/2009) according to age and time of first sample (S1) collection, CoPanFlu-RUN cohort, Reunion Island, 2009.

Age group	First sample collection time	No. of paired blood samples	Seroconversion rate	*P* value
<20 years	W30–31	57	63.2%	<0.0001
	W32–39	325	23.4%	
	W40–44	76	6.6%	
	Total (W30–44)	458	25.5%	
20–59 years	W30–31	132	39.4%	<0.0001
	W32–39	639	15.6%	
	W40–44	156	5.1%	
	Total (W30–44)	927	17.3%	
≥60 years	W30–31	60	16.7%	NA
	W32–39	210	7.1%	
	W40–44	32	0.0%	
	Total (W30–44)	302	8.3%	
All ages	W30–31	249	45.2% (38.0% to 52.3%)	-
	W32–39	1,174	17.4% (14.7% to 20.0%)	
	W40–44	264	5.0% (1.8% to 8.3%)	
	Total (W30–44)	1,687	19.2% (16.9% to 21.4%)	

Data are numbers, percentages (95% confidence intervals) and ALR parameter test *P* value for comparison of seroconversion proportions according to time of first sample (S1) collection at inclusion, in each age group, after controlling for household selection. In the group “All ages”, rates of seroconversion were standardized according to age structure of the community. NA: not assessed. Seroconversion was defined as a shift from seronegative at inclusion (i.e. HIA titer <1/40) to seropositive on follow-up sample, or as a 4-fold increase of reciprocal HIA titer between first and second paired samples for sera tested seropositive on inclusion (i.e. HIA titer ≥1/40). W30–31: first sample collected in early epidemic phase (baseline-proxy); W32–39: first sample collected during the full development of the epidemic wave; W40–44: first sample collected in the immediate post- epidemic phase; W30–44: whole inclusion period.

**Figure 4:**
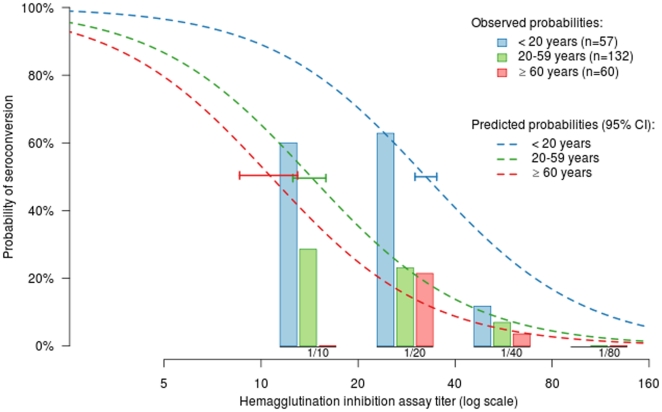
Probability of seroconversion according to age and to baseline pre-epidemic HIA titer.

We then tested in this particular group, the impact of (baseline) pre-epidemic cross reactive antibodies on the rate of seroconversion to pH1N1/2009 (Table 4). No subject with HIA titer superior to 1/40 had evidence of seroconversion to pH1N1/2009. The seroconversion rate in individuals with a HIA titer equal to 1/40 was linked with age, being more important in children and adolescents (<20 yrs). The highest seroconversion rate (>56%) was registered in subjects with HIA titers inferior to 1/40, particularly for the under 20 years where it reached 85%. Hence, the risk of seroconversion decreased when pre-epidemic HIA titer was high after controlling for age (*P*<0.0001) (Figure 4). The multivariate adjusted odds ratio for seroconversion were 0.15 (95%CI: 0.06–0.37, *P*<0.0001) per two-fold increase in baseline titer, 1.79 (95%CI: 1.23–2.59, *P*<0.003) per other household members who seroconverted, 5.33 (95%CI: 1.56–19.27, *P*<0.008) for age <20 years (vs age ≥60 years) and 11.35 (95%CI: 0.41–4.47, *P* = 0.62) for age 20–60 years (vs age ≥60 years). The observed and predicted seroconversion rates according to age and baseline HIA titer are displayed Figure 4.

**Table 4:** Seroconversion rates according to age and baseline-proxy HIA titers in 249 individuals enrolled in pre-pandemic phase, CoPanFlu-RUN cohort, Reunion Island, 2009.

Age group	Baseline-proxy HIA* titer (W30–31)	No. of paired blood samples	Seroconversion rate	*P* value
<20 years	<1/40	40	85.0%	0.0002
	1/40	17	11.8%	
	≥1/80	0	0.0%	
20–59 years	<1/40	85	57.6%	<0.0001
	1/40	43	7.0%	
	≥1/80	4	0.0%	
≥60 years	<1/40	16	56.2%	0.0010
	1/40	28	3.6%	
	≥1/80	16	0.0%	
All ages	<1/40	141	67.0% (58.4% to 75.7%)	-
	= 1/40	88	8.3% (2.2% to 14.4%)	
	≥1/80	20	0.0%	

Data are numbers, percentages (100 * number seroconverters/number tested (95% confidence intervals)) and ALR parameter test *P* value for comparison of seroconversion proportions between baseline-proxy HIA titer (<1/40 versus ≥1/40), in each age, after controlling for household selection. In the group “All ages” seroconversion rates were standardized according to age structure of the community.

*HIA titer: hemagglutination inhibition assay titer. Seroconversion was defined as a shift from seronegative at inclusion (i.e. HIA titer <1/40) to seropositive on follow-up sample, or as a 4-fold increase of reciprocal HIA titer between first and second paired samples for sera tested seropositive on inclusion (i.e. HIA titer ≥1/40).

Finally, we considered the 46 subjects who had been infected by the pandemic virus over the course of the study, verified by a positive qRT-PCR nasal swab, and for whom paired sera were available. Initial HIA antibody titers in this group were <1/40, 1/40, 1/80 and 1/160, in 31 (67.4%), 13 (28.3%), one (2.1%) and one (2.1%) subjects respectively. At the end of the survey, 43 individuals out of 46 (93.5%) were tested seropositive at HIA titer ≥1/40, and 39 (90.7%) at HIA titer ≥1/80. Thirty-four subjects (73.9%) had seroconverted.

## Discussion

The CoPanFlu-RUN cohort was set up to conduct a prospective population-based study investigating the herd immunity induced by the 2009 pandemic influenza virus and identifying risk factors for pH1N1/2009v infection from paired sera collected in an entire community. Most works published to date have used either extensive cross-sectional serosurveys on pre- and post-epidemic independent serum samples, the baseline immunity being assessed from stored frozen samples [5], [7], [8], or non representative adult cohorts (military, health care workers, long-stay patients). Antibody titers were measured by HIA using a cut-off value set at 1/40 as classically recommended. This HIA titer at 1/40 is considered protective, i.e. conferring 50% protection against a viral challenge [20]. Our assay has introduced some changes in the experimental protocol compared to the classic one. The use of a non-inactivated viral antigen, i.e. a native virus, with non-denatured epitopes probably allows detection of antibodies to epitopes of the hemagglutinin not detected in the classic HIA test. This can induce slight differences in the sensitivity of detection of cross-reacting antibodies, but this does not modify the kinetics of Ab and the epidemiological evolution of seroprevalence and does not jeopardize the global comparability of serological results. This is confirmed by the fact that our HI assay detected seroprotective antibody titers in 93.5% and gave evidence seroconversion in 73.9% of qRT-PCR confirmed pH1N1/2009 influenza, all figures close to those reported in the literature [5], [21].

We considered that titers of >1/40, in sera collected from individuals enrolled during weeks 30 and 31 were cross reactive antibodies and not de novo antibodies triggered by the pandemic virus and hence used them as a proxy for baseline pre epidemic immunity. Several arguments support this assumption: i) the first case indicating autochthonous transmission in Reunion Island was reported by the epidemiological surveillance department of La Réunion on 21st July (week 30), i.e. the same day when inclusion started in our study cohort; ii) 7 to 15 days are required to develop an antibody response after viral infection; iii) On weeks 30 and 31, the epidemic activity due to the pandemic virus was very low in our study cohort and it became significant only after week 32. Hence, during weeks 30–31, 103 households were recruited and only 2 households reported ILI cases. Nasal swabs collected from these 2 individuals were tested qRT-PCR negative to the pandemic virus whereas one had evidence of coronavirus and rhinovirus using a multiplex RT-PCR to respiratory viruses (H. Pascalis, manuscript in preparation). In contrast, during weeks 32 to 39, 199 individuals belonging to 99 households reported ILI, among whom 60 individuals had documented infection by the pandemic virus.

Our study shows that a substantial proportion of Reunion Island's population had pre-existing immunity to 2009 pandemic influenza virus with the highest baseline-proxy seroprevalence rate observed among adults aged of 60 years or more. Other studies from all continents had also reported high pre-epidemic seropositivity rates among the elderly [5], [6], [8], [22]–[26], though large variations do exist between countries [10], [11], [23], [27], [28]. These cross reactive antibodies have been interpreted as being the residual signature of the remote exposure of these individuals to H1N1 viruses circulating before 1957 [24], [25], [29], [30]. Baseline seropositivity rates that we report in children and in younger adults (i.e. 30%–35%) were notably higher than those reported from other parts of the world [6], [8], [22], [23], [31]–[33]. However one should note that these baseline antibodies were of low titer, just at the level of the HIA threshold (i.e. 1/40). Several factors could have contributed to this comparatively high baseline rates found in our study: i) It may reflect the fact that the HI test used in our study was marginally more sensitive than the classic one [17]; ii) Some individuals may have already been infected with pH1N1/2009 virus at weeks 30 and 31 and may have triggered an antibody response to the virus. This hypothesis seems unlikely in view of the arguments presented above and of a similar high proportion of sera titering HIA = 1/40 among 122 sera from adult patients sent for diagnostic purposes to the Regional Hospital microbiology laboratory, during the first half of 2009 (i.e. before the 2009 pandemic) (data not shown). However we cannot formally exclude this hypothesis in view of a recently reported study from Taiwan [11] that showed evidence of subclinical community transmission with proved seroconversion several weeks before report of the first documented case in the island. A similar conclusion was also drawn from Australia [34]; iii) our serological test might detect cross-reactive antibodies triggered by recent vaccination with trivalent seasonal influenza vaccine as reported [4], [35]–[39]. However, seasonal influenza vaccines were of rather limited use in Reunion Island, especially in children and young adults; iv) Finally the high baseline titers may reflect the infectious history of the individuals to seasonal influenza viruses cross antigenic with pH1N1/2009 virus as recently suggested for seasonal 2007 H1N1 infection [40]. This serosurvey indicates that a large fraction of the Reunion Island population was infected with the pandemic virus. Younger people, have paid the main tribute to the epidemic as almost two thirds show evidence of seroconversion, confirming earlier clinical reports from the island [12] and accumulating reports from other countries [17], [32], [41], [42] and suggesting that school children have likely played the central role in the epidemic diffusion of the pandemic virus. Lower infection rates were found in adults and the lowest rates were recorded in the elderly.

Based on clinical cases reported to the epidemiological surveillance services [12], it was estimated that 66,915 persons in Reunion Island who consulted a physician were infected by the pH1N1/2009 virus during the 9 weeks of the epidemic, giving a cumulative attack rate of 8.26%. Taking into account those who did not consult a physician, the number of symptomatic infected persons was estimated to 104,067 (attack rate: 12.85%). In fact, the attack rate of pH1N1/2009 infection in our serosurvey was about 42%–44% at the peak of the antibody response (i.e., weeks 40–44), a figure which is at least 3 to 4 times higher than rates of infection based on clinical cases The wide gap between the two estimates indicates that a large fraction (almost two thirds) of those who got infected by pH1N1/2009 virus escaped medical detection, probably because they developed mild disease or asymptomatic infection, a further indication of the benign nature of the virus, at least at the community level. In England, Baguelin et al. [43] estimated that the cumulative incidence rates of infection by the pandemic virus in children were 20 to 40 times higher than that estimated from clinical surveillance.

Our study, as others [6], indicates that pre-existing cross reactive antibodies to pH1N1/2009 at titers ≥1/40 prevented from seroconversion in response to the pandemic virus. This level of pre-existing cross reactive immunity likely confers true protection against infection as about two thirds and one third of documented infection (qRT-PCR positive) in our series have occurred in individuals with baseline HIA titers <1/40 and = 1/40 respectively and less than 5% of documented infections occurred in individuals with base line titers >1/40. The protection was effective not only in older adults but also in younger persons. This indicates that protection was conferred not only by baseline cross reactive antibodies triggered by close pH1N1/2009 viruses that circulated before 1957 (as in the elderly), but also by antibodies likely resulting from recent exposure to seasonal influenza epidemics (as shown in younger persons) [40]. The observed seroconversion rates depend on age, after adjusting for baseline pH1N1/2009 titers. The protective role of increasing age might be explained by a stronger cross-immunity in adults and elderly or by a higher exposure of young subjects to the virus during the 2009 epidemic (due to social contacts and mixing patterns). It may also indicate that immune mechanisms other than cross reactive antibodies detected by HIA (i.e. immunity to neuraminidase and conserved T cells epitopes [44] might develop throughout life, providing additional protection from infection or severe disease, especially in the elderly. Interestingly, evidence is seen for a decline in antibody titers, which occurred soon after the passage of the epidemic wave. In paired sera, this decline was significant enough to bring, within a few weeks, almost 27% of sera that tested positive (i.e. HI titers ≥1/40) in the immediate post epidemic phase to levels under the cut-off value in the second serum sample. This decay accounts for the observation that older adults (≥60 yrs) in the study cohort were apparently almost completely spared by the epidemic if one only considers cumulative incidence rates derived from IHA titration on samples 2 (weeks 45–52). In fact, the cumulative incidence rate in older adults measured just after the epidemic peak (i.e. weeks 40–44) was 20.4%. Similar results of early antibody decay were recently reported [10], [45]. More generally, these data show that serosurveys conducted months after passage of the epidemic, likely underestimate the real extent of pH1N1/2009 infection, compared to antibody titration performed earlier, when humoral responses are at their highest level. Whether the decline in antibody titers has functional immunologic consequence to individuals or within the communities warrants further investigation. However, one should note that there was no second epidemic wave in Reunion Island during the subsequent austral winter seasons in 2010 and 2011. Influenza during the 2010 winter was at a level not higher than the usual passages of seasonal flu, though almost two thirds of documented cases in 2010 were also due to pH1N1/2009v [46]. In addition many fewer pandemic virus isolates were noted during the ongoing 2011 austral winter, strongly suggesting that the first epidemic wave had conferred a solid herd immunity, at the community level.

Our study has some limitations. The fact that the epidemic progression coincided with the implementation of the prospective study, we were not able to collect, strictly speaking, pre-epidemic sera from the cohort members. Therefore we used as proxy base line seroprevalence data from individuals recruited at the very beginning of the investigation when the epidemic activity in the cohort was very low. This may overestimate the base line immunity if subclinical community transmission had occurred before the first cases of pH1N1/2009 influenza were reported. Antibodies to the pandemic virus were detected by HIA, a test that has a good specificity but a rather low sensitivity [46]. Hence, the threshold of 1/40 may underestimate the number of infected individuals. However, rates of seroconversion, the serologic gold standard test based on paired sera, likely gave the most accurate picture of the pandemic in at the community level in Reunion Island.
